# Accurate DNA Synthesis Across 8-Oxoadenine by Human PrimPol

**DOI:** 10.3390/ijms26146796

**Published:** 2025-07-16

**Authors:** Elizaveta O. Boldinova, Alexander A. Kruchinin, Polina N. Kamzeeva, Andrey V. Aralov, Alena V. Makarova

**Affiliations:** 1 Institute of Gene Biology, Russian Academy of Sciences, 34/5 Vavilova St., 119334 Moscow, Russia; lizaboldinova@yandex.ru (E.O.B.); kruchinin77@gmail.com (A.A.K.);; 2 National Research Center “Kurchatov Institute”, Kurchatov sq. 1, 123182 Moscow, Russia; 3 Shemyakin-Ovchinnikov Institute of Bioorganic Chemistry, Russian Academy of Sciences, Miklukho-Maklaya 16/10, 117997 Moscow, Russia

**Keywords:** PrimPol, 8-oxoadenine, DNA translesion synthesis

## Abstract

PrimPol is a human DNA primase and DNA polymerase involved in DNA damage tolerance in both nuclei and mitochondria. PrimPol restarts stalled replication forks by synthesizing DNA primers de novo and also possesses DNA translesion activity (TLS activity). PrimPol efficiently and relatively accurately bypasses several DNA lesions including 8-oxoguanine, thymine glycol and 5-formyluracil. In this work, we showed that PrimPol possesses efficient and accurate TLS activity across 8-oxoadenine, another common DNA lesion caused by oxidative stress. The accuracy of PrimPol on DNA with 8-oxoA was significantly higher compared to DNA containing 8-oxoG. Replacement of Mg^2+^ ions with Mn^2+^ stimulated activity of PrimPol on DNA with 8-oxoA and 8-oxoG as well as undamaged A in a sequence-dependent manner by the lesion skipping (or template scrunching) mechanism. Altogether, our data support the idea that PrimPol possesses efficient TLS activity across a wide range of DNA lesions caused by oxidative stress.

## 1. Introduction

Human PrimPol possesses DNA primase and DNA polymerase activities and is present in both the nucleus and mitochondria [1,2,3]. It is involved in DNA damage tolerance by the restarting of replication forks at the sites of DNA damage and non-B DNA structures such as G-quadruplexes [4,5,6,7,8,9]. Cells deficient in PrimPol are sensitive to DNA-damaging agents [3,10,11].

PrimPol also demonstrates DNA translesion activity (TLS activity) and can bypass a variety of small DNA lesions [1,12]. PrimPol is blocked on DNA with bulky *N*^2^-dG adducts [13] and DNA–protein and AP site–peptide cross-links [14,15]**,** but can bypass and incorporate complementary nucleotides opposite the cisplatin GG cross-link [15].

Previously, we and others demonstrated that PrimPol carries out efficient and relatively accurate synthesis past DNA lesions caused by oxidation such as 8-oxoguanine (8-oxoG) and 5-formyluracil [1,12,16]. Substitution of Mg^2+^ with Mn^2+^ ions also stimulated the synthesis on DNA with thymine glycol [12]. Such efficient TLS activity might facilitate PrimPol-mediated repriming on severely damaged DNA, e.g., containing clustered DNA damage.

Along with 8-oxoG, 8-oxoadenine (8-oxoA) is the most abundant oxidative lesion [17,18,19,20] with dual miscoding properties. The number of 8-oxoA lesions ranged from 10 to 50% of 8-oxoG in [19], but was detected at the level of 0.7 lesions per 10^6^ nucleotides, which corresponds to ~2200 lesions per human genome and is comparable with 8-oxoG levels in [20]. It was also shown that the ratio of 8-oxoA to 8-oxoG reaches 1:1 in some cancer cells [21]. These modified bases readily adopt the syn conformation: 8-oxoG forms stable 8-oxoG(syn):A(anti) Hoogsteen mispair while 8-oxoA efficiently forms the 8-oxoA(syn):G(anti) pair [22,23]. The majority of DNA polymerases preferentially incorporate non-complementary dAMP opposite 8-oxoG (the so-called “A-rule”), leading to G:C → T:A transversions [23,24]. While 8-oxoA is not mutagenic in *Escherichia coli* [25], its mutagenic effect was demonstrated in mammalian cells [26,27]. The 8-oxoA lesion placed in the *HRAS* oncogene mutation hotspot sequence stimulated A:T → C:G transversions and A:T → G:C transitions at a relatively high frequency of mutagenesis comparable [26] or 4-fold-reduced [28] in comparison to that caused by 8-oxoG.

In this work, we, for the first time, report the activity of PrimPol on DNA with 8-oxoA in the *HRAS* oncogene mutation hotspot sequence context. We showed that PrimPol accurately bypasses 8-oxoA in vitro. We also analyzed the effect of metal ions and DNA sequence context on the TLS activity of PrimPol on DNA with 8-oxoA and compared these data with those obtained for 8-oxoG.

Altogether, these findings further support the possible role of PrimPol in the replication of DNA with oxidative damage.

## 2. Results

### 2.1. Efficient and Accurate Bypass of 8-oxoA in Reactions in the Presence of Mg^2+^

**Figure 1:**
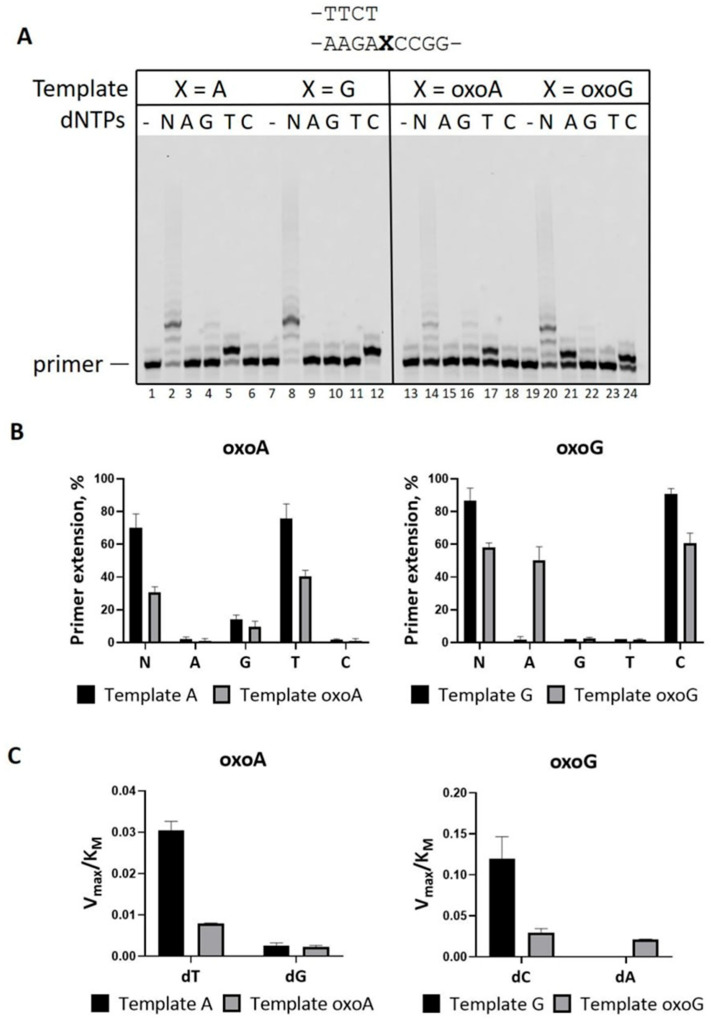
DNA polymerase activity of PrimPol on DNA with 8-oxoA or 8-oxoG in the presence of Mg^2+^. (**A**) Primer extension reactions on DNA containing 8-oxoA or 8-oxoG with Mg^2+^. Reactions were carried out in the presence of 10 mM MgCl_2_, 200 nM PrimPol, 100 nM Cy5-DNA-substrate, and 200 μM of all four dNTPs (N) or individual nucleotide substrates (A—dATP; G—dGTP; T—dTTP; C—dCTP) for 4 min. (**B**) Diagram shows the percent of primer extension on DNA with 8-oxoA or 8-oxoG in reactions in the presence of Mg^2+^ (**A**). The mean values of primer extension and standard errors are indicated. (**C**) Diagram shows the V_MAX_/K_M_ ratio calculated for DNA containing 8-oxoA or 8-oxoG in reactions in the presence of Mg^2+^ (Table 1).

First, we analyzed the activity of PrimPol in reactions in the presence of Mg^2+^ on DNA substrates with the *HRAS* oncogene sequence context 5′-CC**X**AG-3′ containing A, G, 8-oxoA, or 8-oxoG at the mutation hotspot in the +1 position. Both DNA lesions, 8-oxoA and 8-oxoG, only slightly inhibited the activity of PrimPol (Figure 1). Unlike many other DNA polymerases, PrimPol was more accurate and incorporated opposite 8-oxoG complementary dCMP with slight preference over non-complementary dAMP (Figure 1A,B, Table 1). However, PrimPol almost exclusively incorporated complementary dTMP opposite A and 8-oxoA (Figure 1A,B, Table 1). PrimPol incorporated non-complementary dGMP with 3- to 4-fold-reduced efficiency on the DNA template with 8-oxoA and a 10-fold reduction in efficiency on the DNA template with undamaged A compared to dTMP (Figure 1C, Table 1). Interestingly, incorporation of dGMP was observed as a ladder in reactions in the presence of DNA templates containing 8-oxoA, 8-oxoG, or undamaged A but not in reactions with template G (Figure 1A, lanes 4, 10, 16, 22). This activity can be a result of dGMP incorporation opposite C in the +2 and +3 template positions during template scrunching (or lesion skipping), leading to small deletions.

### 2.2. Mn^2+^ Ions Decrease Accuracy of PrimPol on DNA Substrates with 8-Oxopurines

**Figure 2:**
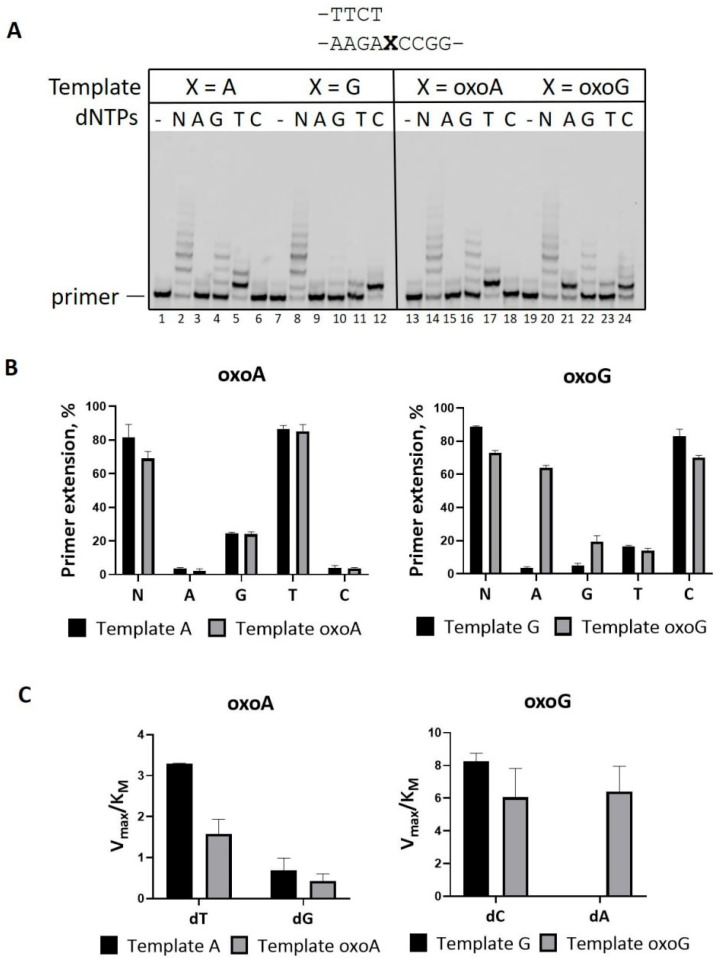
DNA polymerase activity of PrimPol on DNA with 8-oxoA or 8-oxoG in the presence of Mn^2+^. (**A**) Primer extension reactions on DNA containing 8-oxoA or 8-oxoG with Mn^2+^. Reactions were carried out in the presence of 1 mM MnCl_2_, 200 nM PrimPol, 100 nM Cy5-DNA-substrate, and 200 μM of all four dNTPs (N) or individual nucleotide substrates (A—dATP; G—dGTP; T—dTTP; C—dCTP) for 1 min. (**B**) Diagram shows the percent of primer extension on DNA with 8-oxoA or 8-oxoG in reactions in the presence of Mn^2+^ (**A**). The mean values of primer extension and standard errors are indicated. (**C**) Diagram shows the V_MAX_/K_M_ ratio calculated for DNA containing 8-oxoA or 8-oxoG in reactions with Mn^2+^ (Table 1).

The DNA polymerase activity of PrimPol as well as the template scrunching mechanism are stimulated by Mn^2+^ ions [12,13,29]. Indeed, replacement of Mg^2+^ with Mn^2+^ ions stimulated the activity of PrimPol and reduced its accuracy on all DNA templates (Figure 2). PrimPol carried out error-prone synthesis on DNA with 8-oxoG. Enzyme incorporated dAMP slightly more efficient than complementary dCMP (Figure 2A, lane 21, Figure 2C, Table 1).

Mn^2+^ ions also facilitated the incorporation of dGMP and dTMP on DNA with 8-oxoG (Figure 2A, lanes 22 and 23, Figure 2B). Mn^2+^ stimulated the incorporation of dGMP on both templates A and 8-oxoA (Figure 2A, lanes 4 and 16, Table 1). PrimPol was slightly more accurate on DNA with A compared to 8-oxoA in the presence of Mg^2+^ (Table 1). In reactions in the presence of Mn^2+^, PrimPol demonstrated similar accuracy by incorporating dGMP on both templates A and 8-oxoA with 4- to 7-fold-reduced efficiency (Figure 2C, Table 1). The dGMP incorporation was observed as prominent ladders on DNA with A, 8-oxoA, and 8-oxoG, and was likely a result of indels following alternative alignments with a short +2–3 CC template microhomology region.

### 2.3. The Effect of DNA Sequence Context on A and 8-oxoA Bypass

**Figure 3:**
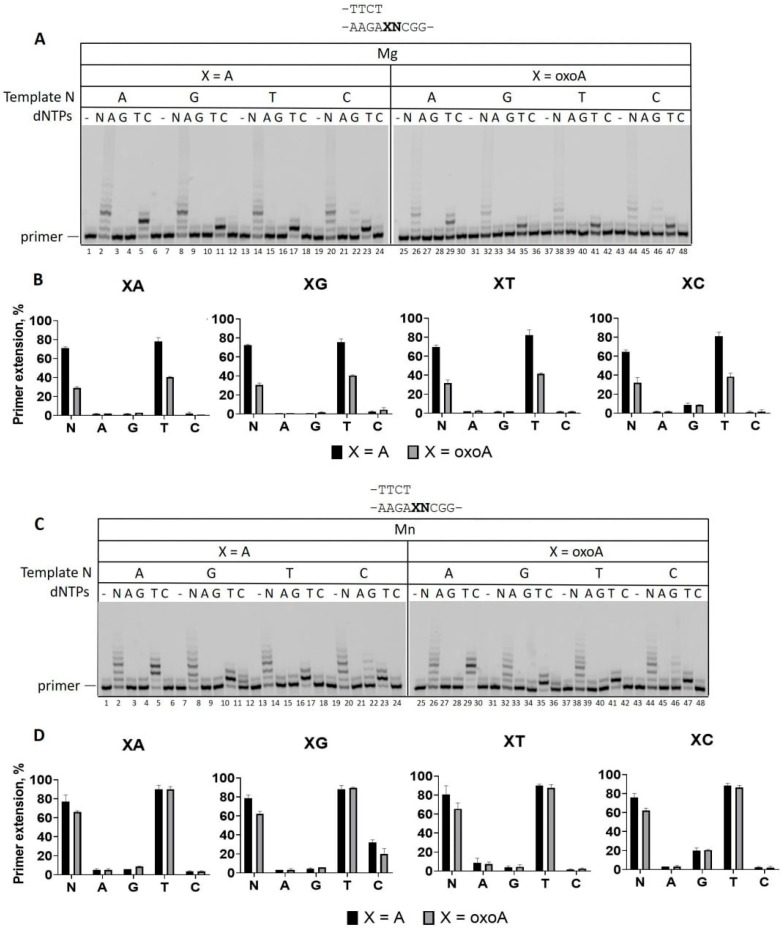
DNA polymerase activity of PrimPol on DNA with A or 8-oxoA in different sequence contexts. (**A**) Primer extension reactions on DNA containing A or 8-oxoA with Mg^2+^. DNA substrates contain A, G, T, or C at the +2 position of the template (N). Reactions were carried out in the presence of 10 mM MgCl_2_, 200 nM PrimPol, 100 nM Cy5-DNA-substrate, and 200 μM of all four dNTPs (N) or individual nucleotide substrates (A—dATP; G—dGTP; T—dTTP; C—dCTP) for 4 min. (**C**) Primer extension reactions on DNA containing A or 8-oxoA with Mn^2+^. DNA substrates contain A, G, T, or C at the +2 position of the template. Reactions were carried out in the presence of 1 mM MnCl_2_, 200 nM PrimPol, 100 nM Cy5-DNA-substrate, and 200 μM of all four dNTPs (N) or individual nucleotide substrates (A—dATP; G—dGTP; T—dTTP; C—dCTP) for 1 min. (**B**,**D**) Diagrams show the percent of primer extension on DNA with A or 8-oxoA in reactions in the presence of Mg^2+^ (**A**) and Mn^2+^ (**C**). The mean values of primer extension and standard errors are indicated.

To test the effect of DNA sequence context on nucleotide incorporation, we replaced C in the +2 position of the *HRAS* oncogene sequence with A, G, or T. All replacements increased the accuracy of PrimPol and reduced the incorporation of dGMP on DNA substrates with a template containing undamaged A or 8-oxoA lesion in the presence of Mg^2+^ (Figure 3A,B). In reaction with Mn^2+^, PrimPol also incorporated dGMP on DNA templates with A and 8-oxoA in the *HRAS* 5′-CC**X**AG-3′ sequence context only (Figure 3C,D). Moreover, PrimPol incorporated dCMP on both A- and 8-oxoA-containing DNA substrates after replacement of the +2 template C with G. Altogether, these data suggest that PrimPol carries out efficient and accurate DNA synthesis across 8-oxoA. PrimPol also induces microdeletions with low efficiency in a sequence-dependent manner.

## 3. Discussion

8-oxoG and 8-oxoA are the most common lesions caused by reactive oxygen species. 8-oxoG is ambiguously read by DNA polymerases, leading to mutations after the next round of replication rather than showing blocking effects. The available evidence suggests that 8-oxoA in mammalian cells also has a moderate mutagenic potential and induces A:T → C:G transversions and A:T → G:C transitions in the *HRAS* 5′-CCXAG-3′ sequence [26,27,28].

In contrast to 8-oxoG, the activity of eukaryotic DNA polymerases opposite 8-oxoA remains poorly characterized. Only three DNA polymerases, namely Pol α, Pol β, and Pol η, have been studied to date [26,30,31]. These DNA polymerases preferentially incorporated opposite 8-oxoA dTMP and small amounts of dGMP. Pol α carried out quite accurate DNA synthesis by incorporating complementary dTMP 10-fold more efficiently compared to non-complementary dGMP [30], while translesion Pol η incorporated dTMP and dGMP with almost similar efficiency [31]. The accuracy of Pol β varied from 4- to 18-fold preference for dTMP over dGMP on DNA substrates with different sequence contexts [30,31].

Human PrimPol is a unique DNA primase involved in DNA damage tolerance pathways in both nuclei and mitochondria [1,16,32]. PrimPol can encounter DNA lesions during repriming events and also possesses DNA translesion activity. PrimPol is also known for its template scrunching activity, which can generate small deletions [29,33]. This activity is stimulated by Mn^2+^ and DNA lesions (lesion skipping mechanism) [12,29].

In this work, we studied PrimPol bypass of 8-oxopurine lesions in the *HRAS* sequence context. We demonstrated that PrimPol bypasses 8-oxoA with high efficiency and relatively high fidelity. PrimPol preferentially incorporated complementary dTMP opposite 8-oxoA in reactions in the presence of both Mg^2+^ and Mn^2+^ cofactors. PrimPol also incorporated dGMP on DNA substrates with 8-oxoA with 3- to 4-fold-reduced efficiency compared to the complementary dTMP. Unlike other DNA polymerases, dGMP incorporation by PrimPol was observed on DNA with both 8-oxoA and undamaged A. It was sequence-dependent and was stimulated by short CC nucleotide repeats in the *HRAS* **CC**XAG sequence. Replacement of C in the +2 template position abrogated dGMP incorporation, suggesting that it is mediated by the template scrunching mechanism and causes deletions.

The error-prone dGMP incorporation was observed in reactions with both Me^2+^ cofactors but the efficiency of dGMP incorporation was higher with Mn^2+^. In particular, PrimPol incorporated dGMP on DNA substrates with 8-oxoA ~3-fold less efficiently compared to undamaged A in the presence of Mg^2+^ ions and with equal efficiencies for both templates in reactions with Mn^2+^. Another type of error—dCMP incorporation on DNA templates with A or 8-oxoA—was exclusively stimulated by Mn^2+^ ions and was observed only in reactions with the C**G**XAG sequence context and guided by G in the +2 templates position. Altogether, these data are in agreement that PrimPol misincorporates nucleotides in a sequence-dependent manner utilizing the Mn^2+^-stimulated template scrunching mechanism, which is not specific to the 8-oxoA lesion. Our data also suggest that the rate of PrimPol-mediated errors is relatively low in all tested sequence contexts and PrimPol is unlikely to contribute to the 8-oxoA-induced mutagenesis in living cells.

The 5′-flanking nucleotide near 8-oxoG affects the accuracy of TLS by Pol η [34]. Interestingly, in our work (in the *HRAS* oncogene sequence context 3′-GA**8**-**oxoG**CC-5′), PrimPol demonstrated lower accuracy on DNA with 8-oxoG than in previous studies and incorporated dCMP and dAMP with almost equal efficiencies. In contrast, PrimPol incorporated dCMP almost exclusively or about 6- to 8-fold more efficiently than non-complementary dAMP in other sequence contexts such as 3′- CG**8-oxoG**CA-5′ [1], 3′-AG**8**-**oxoG**CA-5′ [35], 3′-TG**8**-**oxoG**AC-5′ [12]**,** and 3′-AG**8**-**oxoG**TT-5′ [16]. Crystallographic PrimPol studies demonstrated that 8-oxoG in DNA containing the 3′-GC**8**-**oxoG**AC-5′ sequence in complex with both incoming dCTP and dATP adopts the *anti* or *syn* conformation, respectively, without significant structural hindrance within the active site, which supports the relatively low accuracy of PrimPol opposite 8-oxoG [36]. Our results corroborate these observations. It is possible that stacking interactions of 8-oxoG with flanking nucleobases contribute to its positioning in the *anti* or *syn* conformation.

Since PrimPol efficiently bypasses several DNA lesions including 8-oxoA, 8-oxoG, 5-fU, and thymine glycol, we suggest that PrimPol carries out efficient TLS across a wide range of DNA lesions caused by oxidative stress. Indeed, PrimPol attenuates the response of A549 cells to oxidative damage [37]. Also, PrimPol as a component of the MUS81-LIG4 axis takes part in replication fork restart during transcription-dependent replication stress under excessive reactive oxygen species action [38]. A recent study demonstrated that PrimPol can contribute to an SBS-A mutational signature resembling the mutagenic effect of 8-oxoG due to its ability to bypass oxidized damage [39].

## 4. Materials and Methods

### 4.1. DNA Templates and Enzymes

PrimPol was purified from *E. coli* as described in [15]. DNA oligonucleotides used in this study (Table 2) were synthetized as described previously [40]. 8-oxoA and 8-oxoG lesions were placed in a sequence context similar to the *HRAS* CCXAG mutagenesis hot spot [26] in TemplateXA. The templates TemplateXG, TemplateXT, and TemplateXC differ from TemplateXA by the single substitution at the +2 position. To prepare DNA substrates, the 5′-Cy5-labelled primer Pr18-Cy5 of ^32^P-labelled primer Pr18 was annealed to the corresponding unlabeled template oligonucleotides at a molar ratio of 1:1.1 in 100 mM NaCl by heating to 97 °C and slowly cooling to 4 °C.

### 4.2. DNA Polymerase Reactions for the Primer Extension Assay

Primer extension reactions were performed in 20 µL containing 100 nM DNA substrate, 200 μM dNTP, 30 mM HEPES pH 7.0, 10 mM MgCl_2_ or 1 mM MnCl_2_, 100 µg/mL BSA, 1 mM DTT, 4% glycerol, and 100–200 nM PrimPol. Reactions were incubated at 37 °C for 1–4 min, stopped by the addition of an equal volume of 2× loading buffer (20 mM EDTA, 0.001% bromophenol blue, 96% formamide), and heated for 5 min at 95 °C. The reaction products were resolved on 21% polyacrylamide gels with 8 M urea, visualized on Typhoon 9400 (GE Healthcare, Chicago, IL, USA), and analyzed with ImageQuant software v8.2. All experiments were repeated three times. The percent of the extended primer (PrExt) was calculated for each reaction, and the mean values of PrExt with the standard errors are shown in figures.

### 4.3. Steady-State Kinetics Analysis of dNMP Incorporation

To quantify the incorporation of individual dNMPs opposite DNA lesions, we varied the dNTP concentration from 2.5 to 6000 μM in reactions in the presence of 10 nM PrimPol and 10 mM MgCl_2_ and from 0.25 to 1000 µM in reactions with 5 nM PrimPol and 1 mM MnCl_2_. Depending on the lesion, the reactions were incubated for 3–20 min with MgCl_2_ and for 1–10 min with MnCl_2_ to ensure that less than 40% of the primer was utilized. Calculations were performed using GraFit 5 software (Erithacus Software, East Grinstead, UK). The data were fit to the Michaelis–Menten equation V = V_max_ × [dNTP])/(K_M_ + [dNTP]), where V and V_max_ are the observed and maximum rates of the reaction (in percentages of utilized primer per minute), respectively, and K_M_ is the apparent Michaelis constant.

## Figures and Tables

**Table 1:** Steady-state kinetics analysis of dNMP incorporation opposite A, G, 8-oxoA, and 8-oxoG.

Template	dNMP	V_max_, % per Min	K_M_, μM	V_max_/K_M_	F_inc_ **
**Mg^2+^**					
**Template A**	dT^radio^ *	12.9 ± 0.7	420 ± 37	0.031 ±0.003	
	**dT**	12.2 ± 0.7	400 ± 42	0.031 ± 0.002	1
	**dG**	0.4 ± 0.004	184 ± 46	0.003 ± 0.0005	0.09
**Template oxoA**	**dT^radio^**	3.6 ±0.3	610 ± 11	0.006 ±0.001	
	**dT**	5.3 ± 0.1	677 ± 16	0.008 ± 0.0001	1
	**dG**	0.4 ± 0.005	182 ± 33	0.002 ± 0.0001	0.25
**Template G**	**dC**	9 ± 0.9	73 ± 5	0.123 ± 0.019	
	**dA**	ND ***			
**Template oxoG**	**dC**	3.8 ± 0.3	128 ± 3	0.029 ± 0.004	1
	**dA**	2.9 ± 0.3	137 ± 9	0.021 ± 0.0005	0.72
**Mn^2+^**					
**Template A**	**dT^radio^**	50 ± 1.5	11 ± 1	4.5 ± 0.3	
	**dT**	40 ± 0.9	12.2 ± 0.3	3.3 ± 0.01	1
	**dG**	2.7 ± 0.5	4 ± 0.3	0.7 ± 0.2	0.2
**Template oxoA**	**dT^radio^**	28 ± 1.4	10 ± 0.5	2.7 ± 0.1	
	**dT**	22.5 ± 0.02	14.6 ± 1.9	1.6 ± 0.2	1
	**dG**	2.4 ± 0.2	3.9 ± 0.8	0.4 ± 0.1	0.25
**Template G**	**dC**	41.5 ± 0.1	5.1 ± 0.2	8.2 ± 0.3	
	**dA**	ND			
**Template oxoG**	**dC**	23.5 ± 1	4 ± 0.5	6 ± 1	1
	**dA**	15.9 ± 0.6	2.5 ± 0.3	6.4 ± 0.9	1.1

* dT^radio^—data calculated for ^32^P-labelled DNA substrate. ** F_inc_ = V_max_^non-complementary^/K_M_^non-complementary^/V_max_^complementary^/K_M_^complementary^. *** ND—not detected.

**Table 2:** Oligonucleotides used in this study.

Oligonucleotide	Sequence 5′-3′
Pr18-Cy5	Cy5-AGGGCAGAGTATTCTTCT
Pr18	AGGGCAGAGTATTCTTCT
TemplateXA	TTTTTTTTTTTTTTTTTTTTTTTTTTTTTTTTTTTTTTTATACCGCAGGCA**X**AGAAGAATACTCTGCCCT
TemplateXG	TTTTTTTTTTTTTTTTTTTTTTTTTTTTTTTTTTTTTTTATACCGCAGGCG**X**AGAAGAATACTCTGCCCT
TemplateXT	TTTTTTTTTTTTTTTTTTTTTTTTTTTTTTTTTTTTTTTATACCGCAGGCT**X**AGAAGAATACTCTGCCCT
TemplateXC	TTTTTTTTTTTTTTTTTTTTTTTTTTTTTTTTTTTTTTTATACCGCAGGCC**X**AGAAGAATACTCTGCCCT

X = A, 8-oxoA, G, or 8-oxoG.

## Data Availability

All data are available upon request.

## Abbreviations

The following abbreviations are used in this manuscript:

8-oxoA	8-oxoadenine
8-oxoG	8-oxoguanine
